# Impact of Voluntary Muscle Activation on Stretch Reflex Excitability in Individuals With Hemiparetic Stroke

**DOI:** 10.3389/fneur.2022.764650

**Published:** 2022-03-08

**Authors:** Jacqueline R. Patterson, Julius P. A. Dewald, Justin M. Drogos, Netta Gurari

**Affiliations:** ^1^Department of Physical Therapy and Human Movement Sciences, Northwestern University, Chicago, IL, United States; ^2^Northwestern University Interdepartmental Neuroscience, Northwestern University, Chicago, IL, United States; ^3^Department of Physiology, Northwestern University, Chicago, IL, United States; ^4^Department of Biomedical Engineering, Northwestern University, Evanston, IL, United States; ^5^Department of Mechanical Engineering, Northwestern University, Evanston, IL, United States

**Keywords:** stroke, hypertonia, spasticity, motoneuron, stretch reflex, stretching, robotics

## Abstract

**Objective:**

To characterize how, following a stretch-induced attenuation, volitional muscle activation impacts stretch reflex activity in individuals with stroke.

**Methods:**

A robotic device rotated the paretic elbow of individuals with hemiparetic stroke from 70° to 150°, and then back to 70° elbow flexion at an angular speed of 120°/s. This stretching sequence was repeated 20 times. Subsequently, participants volitionally activated their elbow musculature or rested. Finally, the stretching sequence was repeated another 20 times. The flexors' stretch reflex activity was quantified as the net torque measured at 135°.

**Results:**

Data from 15 participants indicated that the stretching sequence attenuated the flexion torque (*p* < 0.001) and resting sustained the attenuation (*p* = 1.000). Contrastingly, based on data from 14 participants, voluntary muscle activation increased the flexion torque (*p* < 0.001) to an initial pre-stretch torque magnitude (*p* = 1.000).

**Conclusions:**

Stretch reflex attenuation induced by repeated fast stretches may be nullified when individuals post-stroke volitionally activate their muscles. In contrast, resting may enable a sustained reflex attenuation if the individual remains relaxed.

**Significance:**

Stretching is commonly implemented to reduce hyperactive stretch reflexes following a stroke. These findings suggest that stretch reflex accommodation arising from repeated fast stretching may be reversed once an individual volitionally moves their paretic arm.

## Introduction

An estimated 20–40% of survivors of a stroke exhibit hyperactive stretch reflexes, or spasticity, defined as a position- and velocity-dependent resistance to muscle stretch (1–7). Hypertonia and associated spasticity are thought to originate from increased spinal motoneuron excitability (2, 8–13) and are shown to impair mobility, posture, and hygiene (4, 6, 7). One common approach to reduce hyperactive stretch reflexes is to stretch the affected limb (14–17). For example, consecutive stretches fast enough to elicit stretch reflexes, as confirmed by surface electromyography (sEMG), have been shown to induce reflex accommodation when the limb is relaxed (15). Even so, it remains unknown whether the reflex accommodation that arises from stretching is sustained once an individual volitionally activates their muscles.

Evidence indicates that subsequent volitional muscle activation may negate the stretch-induced accommodation. Previous work established that norepinephrine, which alters stretch reflex activity, increases in cats with volitional movement (18). Additional experiments in humans revealed that volitional muscle activation prior to and during a task amplifies stretch reflex responses (12, 19). Combined, these findings indicate that volitional muscle activation increases stretch reflex activity (12, 18, 19). Even so, the question remains whether volitional muscle activation impacts stretch reflex excitability after stretch-induced accommodation.

The current study investigated the impact of voluntary muscle activation on stretch reflex activity following stretch-induced accommodation in individuals with stroke. A protocol that has been shown to accommodate the stretch reflex in the stroke population was used, specifically applying consecutive stretches that induce stretch reflexes to a relaxed arm (15). Research suggests that muscle activation amplifies motoneuron excitability and, in turn, stretch reflex activity (12, 18, 20). Hence, we hypothesized that voluntary muscle activation would increase stretch reflex activity when compared to the accommodated level induced from consecutive stretches.

## Materials and Methods

### Participants

This investigation was approved by the Northwestern University Institutional Review Board and complies with the 1964 Declaration of Helsinki. Participants provided written informed consent and were evaluated by a licensed clinician. Eligibility criteria included: >1 year post-stroke; paresis confined to one side; passive range-of-motion about the paretic elbow between 70° and 150°, with 180° being full extension; volitional control about the paretic elbow in extension and flexion; no use of anti-spastic agents in the previous 6 months; absence of severe cognitive deficits and contractures; and ability to detect a movement at the paretic arm (21).

### Experimental Setup

**Figure 1:**
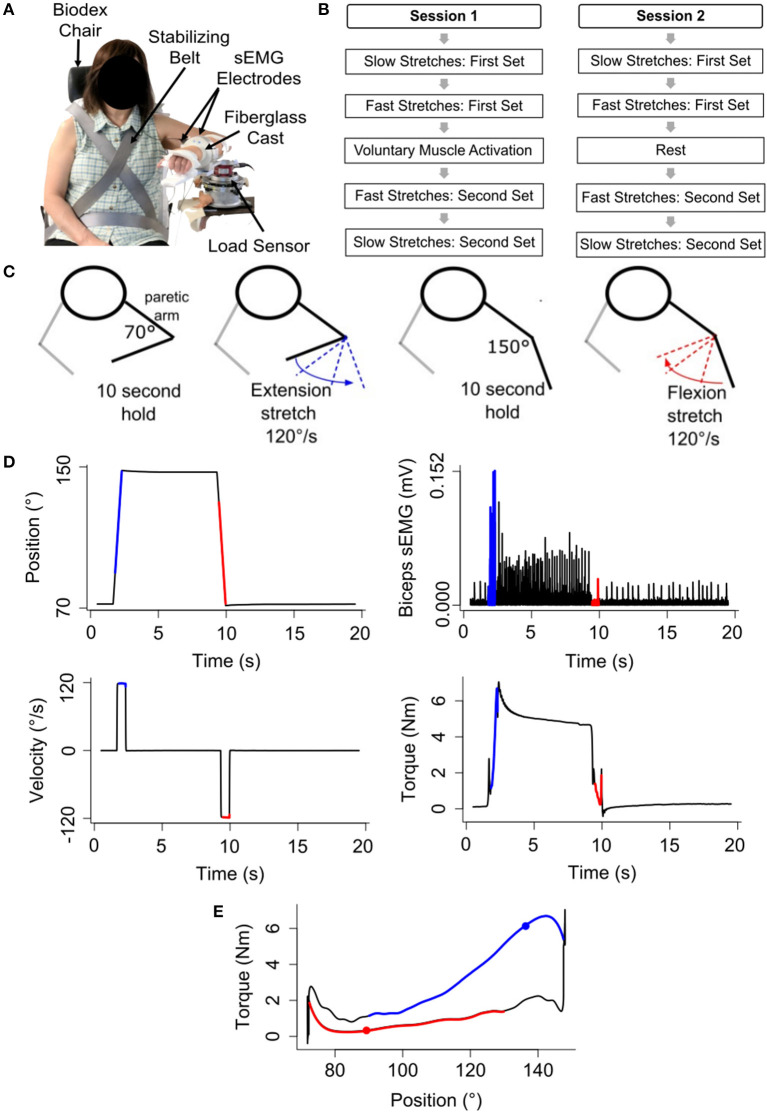
Experimental setup and procedures. **(A)** Participant sitting with their paretic forearm affixed to the robotic device. **(B)** Five tasks of each session. **(C)** Representation of one repetition of the stretching sequence. **(D)** Example angular position, angular velocity, biceps brachii sEMG activity, and torque data during one repetition of the fast stretching sequence. Blue and red lines indicate the constant velocity portion in elbow extension and flexion, respectively. **(E)** Example torque vs. angular position data. The blue filled circle identifies the flexion torque at 135°, and the red filled circle identifies the extension torque at 88°. Blue and red lines indicate the constant velocity portion in elbow extension and flexion, respectively.

Participants sat in a Biodex chair (System 3 Pro^TM^; Shirley, NY, USA) with their trunk stabilized (Figure 1A). Their paretic forearm was fixed to a manipulandum at 85° shoulder abduction and 30° shoulder flexion, and its weight was fully supported. A Harmonic Drive^®^ FHA-17C-100 motor with attached US250 encoder (Peabody, MA, USA) rotated the paretic forearm and measured its angular position with a resolution of 0.000225°. A Futek reaction torque sensor (Model Number TFF600; Irvine, CA, USA) measured torques with a resolution of 0.013Nm. Surface electromyography (sEMG) electrodes (Delsys, 16-channel Bagnoli EMG System; Boston, MA) placed on the muscle bellies quantified activity of the elbow flexors (biceps brachii, brachioradialis) and extensors (triceps brachii lateral head). The software ran at 4 kHz, and data were saved at 1 kHz.

### Procedures

A plethora of stretching approaches exist, which vary in angular range, velocity, repetitions, and frequency (22). The stretching protocol selected for this study was modeled after one protocol that demonstrated stretch reflex accommodation in the stroke population (15); this approach is commonly used in quantitative ramp stretching protocols (3, 19, 23). Using this protocol, participants completed a 2 h session on two separate days (Figures 1B,C).

1. Slow Stretches: First Set

The paretic forearm was extended from 70° to 150°, held for 10 s, flexed from 150° to 70°, and held for 10 s. This stretching sequence was repeated five times at an angular speed of 6°/s to avoid stretch reflex activity, as confirmed with sEMG. In turn, we could quantify passive musculoskeletal properties (15, 24).

2. Fast Stretches: First Set

The stretching sequence described above was repeated 20 times at an angular speed of 120°/s to evoke flexor and extensor stretch reflex activity, as determined with sEMG (3, 15, 24) (Figure 1D).

3. Testing Condition

Voluntary Muscle Activation: During the first session, participants extended and flexed about their paretic elbow through their active range-of-motion five times as quickly as possible.

Rest: This session was included to determine whether the time elapsed, rather than the voluntary muscle activation, elicited a change in the stretch reflex activity. Participants rested at 70° elbow flexion for an equal duration to the time that elapsed between the first set and second set of 120°/s stretches for the voluntary muscle activation session.

4. Fast Stretches: Second Set

The stretching sequence was repeated 20 times at 120°/s.

5. Slow Stretches: Second Set

The stretching sequence was repeated five times at 6°/s.

### Data Analyses

#### Quantifying Passive Musculoskeletal Stiffness—Slow Stretches

We aimed to quantify the passive musculoskeletal stiffness at the beginning and end of each session. This was achieved by analyzing the data acquired during the slow stretches, which were implemented to avoid stretch reflex activity as determined by an absence of sEMG activity. Data were removed for a participant's entire session if the muscle activity during these slow stretches was not deemed quiescent, based on the sEMG data. Since the stiffness profile is nonlinear for the range of angles that we tested, we used a proxy measure to describe the passive musculoskeletal stiffness. Specifically, we quantified the difference in the mean torque when the forearm was held at two discrete positions, 150° and 70° (τ_150°_ − τ_70°_) Using this approach, we could ensure that the difference in the mean torque was based on consistent angular positions for all participants, permitting this outcome measure to be a good proxy for passive musculoskeletal stiffness.

#### Quantifying Stretch Reflex Activity—Fast Stretches

##### Modeling

We used the following two outcome measures so that subsequent analyses could identify changes in reflex activity: (1) net torque for any stretch, *i*, and (2) change in net torque between any two stretches, *i* and *j* (not necessarily consecutive).

To begin, we modeled the net torque (**τ**_*Net*_) about the elbow for every 120°/s stretch in extension or flexion as a summation of the neural stretch reflex (**τ**_*Reflex*_) component and passive musculoskeletal inertial (**τ**_*Inertia*_), damping (**τ**_*Damping*_), and stiffness (**τ**_*Stiffness*_) components:


(1) $$\begin{array}{l} \tau_{Net}\left(\theta \left(t\right)\right)=\tau_{Inertia}\left(\theta \left(t\right)\right)+\;\tau_{Damping}\left(\theta \left(t\right)\right)+\tau_{Stiffness}\left(\theta \left(t\right)\right)\\ \text{ ​ }+\tau_{Reflex}\left(\theta \left(t\right)\right)\text{​}. \end{array}$$

θ and *t* indicate angular position and time.

To avoid the influence of the passive musculoskeletal inertial and damping components, we extracted and analyzed torque data during the constant velocity portion of each stretch. Since the robotic device implemented each stretch using the same control algorithm, the passive musculoskeletal inertial and damping components can be assumed to be comparable within a testing session for each participant such that for any two stretches, *i* and *j*:


(2) $$\begin{array}{l} \tau_{Inertia_{i}}\left(\theta \left(t\right)\right)-\tau_{Inertia_{j}}\left(\theta \left(t\right)\right)=0\text{ and }\\ \tau_{Damping_{i}}\left(\theta \left(t\right)\right)-\tau_{Damping_{j}}\left(\theta \left(t\right)\right)=0. \end{array}$$

Prior research indicates that the passive musculoskeletal stiffness can be modified with stretching, particularly when using much longer stretching durations than those used in our study (25–29). Whether changes in passive musculoskeletal stiffness occur for the shorter stretching duration used in our protocol remains unknown (28). As such, analyses were needed to determine whether the passive musculoskeletal stiffness did change due to the 120°/s stretches used in our protocol. We refer the reader to sections Quantifying Passive Musculoskeletal Stiffness—Slow Stretches, Analysis of Passive Musculoskeletal Stiffness—Slow Stretches, and Passive Musculoskeletal Stiffness for information regarding how we obtained our outcome measures for the passive musculoskeletal stiffness and, subsequently, ran our analyses. If the passive musculoskeletal stiffness was not found to significantly change within one testing session, we could conclude that the passive musculoskeletal stiffness for any two stretches, *i* and *j*, within that testing session was comparable such that the difference between them would cancel one another out:


(3) $$\tau_{Stiffness_{i}}\left(\theta \left(t\right)\right)-\tau_{Stiffness_{j}}\left(\theta \left(t\right)\right)=0.$$

Therefore, a comparison of the net torque for any two stretches, *i* and *j*, within one testing session becomes a comparison of solely the reflex component.

While we can use the logic provided above to indicate that the passive musculoskeletal inertial, damping, and stiffness components remain comparable within a testing session, the same assumption cannot be made between testing sessions. The measurements obtained for the passive musculoskeletal inertial, damping, and stiffness components of Equation (1) may not be comparable between testing sessions due to day-to-day measurement errors related to the set up of the participant. To address this limitation, the outcome measure used to compare changes in the reflex activity between testing sessions was the change in the net torque between any two stretches, *i* and *j*, within one testing session:


(4) $$\begin{array}{l} \tau_{Diff_{\left(i,j\right)}}\left(\theta \left(t\right)\right)=(\tau_{Inertia_{i}}\left(\theta \left(t\right)\right)+\;\tau_{Damping_{i}}\left(\theta \left(t\right)\right)\\ \;+\tau_{Stiffness_{i}}\left(\theta \left(t\right)\right)+\tau_{Reflex_{i}}\left(\theta \left(t\right)\right))-(\tau_{Inertia_{j}}\left(\theta \left(t\right)\right)\\ \;+\tau_{Damping_{j}}\left(\theta \left(t\right)\right)+\tau_{Stiffness_{j}}\left(\theta \left(t\right)\right)+\tau_{Reflex_{j}}\left(\theta \left(t\right)\right)). \end{array}$$

If the passive musculoskeletal inertial, damping, and stiffness components remain similar within a single testing session, as discussed above, then the change in the net torque between the two stretches, *i* and *j*, simplifies to the change in the stretch reflex activity:


(5) $$\tau_{Diff_{\left(i,j\right)}}\left(\theta \left(t\right)\right)=\;\tau_{Reflex_{i}}\left(\theta \left(t\right)\right)-\;\tau_{Reflex_{j}}\left(\theta \left(t\right)\right).$$

As such, the change in net torque can be used to compare the impact of volitional muscle activation vs. rest on changes in stretch reflex activity.

##### Reflex Elicitation

Data related to the flexors and/or extensors were removed for a participant's session if the muscle activity was deemed insufficient, based on sEMG data, during the first stretch of the first set of 120°/s stretches.

##### Torque Extraction

The torque data were filtered using a forward-backward low-pass filter with a 5 Hz cut-off frequency (3, 12, 25, 30, 31). Following, torque values at the angles of 135° and 88° were extracted during each stretch in extension and flexion, respectively, to quantify stretch reflex activity of the flexor and extensor muscles, respectively (Figure 1E). These empirically selected angles permitted consistent extraction of torque responses across all participants during the constant velocity portion of each stretch.

##### Short- and Long-Latency Response

In addition to examining the torque response, we analyzed the participants' sEMG data to identify the short-latency response, arising from spinal-cord circuitry, and long-latency response, potentially involving cortical circuitry. We extracted the sEMG data at time points corresponding to the response time from the spinal (20–50 ms) and transcortical (50–150 ms) components of the stretch reflex (19). The relevant sEMG data were first prepared for these analyses by subtracting the mean sEMG activity from each movement so that the signal had a mean value of zero. Following, the signal was rectified and then smoothed using a low-pass forward-backward filter with a cutoff frequency of 5 Hz. Next, the baseline, pre-activation signal was determined by taking the mean of this processed sEMG signal for the 500 ms prior to the movement, and averaging it across every stretch in extension and flexion, respectively, within a 120°/s stretching set. The corresponding pre-activation signal prior to the movement in extension or flexion was removed from the average sEMG signal between 20 and 50 ms and between 50 and 150 ms, respectively, of every stretch within that 120°/s stretching set to give the short-latency response (SLR) and long-latency response (LLR) for the flexors and extensors.

We highlight that the LLR is comprised of both transcortical and spinal components since the sEMG spinal component of the stretch reflex is present from ~20 ms post perturbation initiation. Thus, to deduce contributions arising from the transcortical component, we needed to confirm that the spinal component was comparable during the measurements occurring at 20–50 ms (SLR) and 50–150 ms (LLR) after perturbation initiation. The spinal component would only be comparable if the angular speed at which the forearm rotated was the same during each of these respective time windows. If the angular speed of rotation was not comparable, then we would not be able to deduce whether changes from the SLR to the LLR arise due to changes in the spinal vs. the transcortical component. For this reason, we report the mean angular speed of rotation of the forearm during the SLR and LLR and summarize our results accordingly.

### Statistical Analyses

#### Analysis of Passive Musculoskeletal Stiffness—Slow Stretches

We aimed to indicate whether the passive musculoskeletal stiffness remained consistent within a single testing session. This was achieved by using a pairwise *t*-test to determine whether, across all participants, the difference in the mean torque (τ_150°_ − τ_70°_), as defined in section Quantifying Passive Musculoskeletal Stiffness—Slow Stretches, significantly changed between the first and second set of slow stretches for a single testing session. If the difference in the mean torque was found to significantly change, then the proposed analyses in section Quantifying Stretch Reflex Activity—Fast Stretches would not hold.

#### Analysis of Stretch Reflex Activity—Fast Stretches

##### Impact Across All Stretches

We aimed to indicate whether the torque arising due to stretch reflex activity was impacted by the fast 120°/s stretches before and after volitional muscle activation and rest. To do so, the net torque outcome measure, defined in section Quantifying Stretch Reflex Activity—Fast Stretches, was fit to a linear mixed-effects model, with participant as a random effect. We identified whether the net torque depended on the stretch repetition (1–20) and set (first, second) for each testing condition (volitional muscle activation, rest) and muscle group (flexors, extensors). An analysis of variance with a Tukey adjustment identified significant fixed effects.

##### Impact Across Pairs of Stretches

In addition to identifying effects across all stretches, we also identified effects for specific pairs of stretches. For these pairs, data were analyzed across all participants using a pairwise *t-*test with a Bonferroni correction.

To begin, we indicated whether the net torque significantly differed between the following pairs of stretches for each testing condition and muscle group. We compared the first set's final stretch and second set's first stretch to determine whether volitional muscle activation and rest had an immediate effect on stretch-induced accommodated reflex activity. Additionally, we compared the first stretch of the first set to the first stretch of the second set to determine the impact of volitional muscle activation and rest on reflex activity when compared to the reflex activity prior to stretching. Moreover, we compared the final stretch of the first set to the final stretch of the second set to determine whether volitional muscle activation and rest affected the extent to which the reflex could be accommodated with the fast 120°/s stretches.

Following, for each muscle group we determined whether the difference in the net torque between each of these pairs of stretches within each session depended on the testing condition.

##### Short- and Long-Latency Response

We aimed to indicate whether the short- and/or long-latency response corresponding to spinal reflexes and potentially cortical circuitry, respectively, were affected by the fast 120°/s stretching after the participant volitionally activated their muscles or relaxed. To do so, we fit the SLR and LLR outcome measures described in section Quantifying Stretch Reflex Activity—Fast Stretches to a linear mixed-effects model, with participant as a random effect. Subsequently, we determined whether the SLR and LLR depended on the stretch repetition (1–20) and set (first, second) for each testing condition (volitional muscle activation, rest) and muscle group (flexors, extensors). An analysis of variance with a Tukey adjustment identified significant fixed effects.

## Results

### Participants

Seventeen individuals 12 ± 9 (μ ± σ) years post-stroke participated (Table 1). Participants had upper-extremity Fugl-Meyer motor assessment (UE FMA) scores spanning 12–48 (μ ± σ UE FMA: 28 ± 11).

**Table 1:** Study participants.

**Participant**	**Gender**	**Age**	**Years Post Stroke**	**Lesion Location**	**UE FMA Score**	**Biceps Brachii MAS**
1	M	70	14	R: Fr,BG,I	29	1+
2	M	51	29	NA	26	1+
3	M	61	3	R: IC	39	1
4	M	70	22	L: Th,IC,BG,T,I	12	2
5	M	47	10	R: Th,IC,BG	18	2
6	F	69	14	L: Th,IC,BG	12	2
7	F	66	32	L: Th,IC,BG	16	1+
8	M	48	13	R: Th,IC,BG	38	2
9	M	43	4	L: Th,IC,BG,Fr,P	39	1+
10	M	63	14	L: IC	48	1+
11	M	59	7	L: IC	22	3
12	M	73	15	NA	27	1+
13	M	48	7	R: Th,BG	33	1+
14	F	49	11	R: IC,Fr,P	28	1+
15	M	64	8	R: IC	17	1+
16	M	62	4	NA	36	2
17	M	58	1	NA	NA	1+

*UE-FMA, upper-extremity score of Fugl-Meyer motor assessment (max = 66); MAS, modified Ashworth scale score (max = 4); M, male; F, female; R, right; L, left; Th, thalamus; IC, internal capsule; BG, basal ganglia; Fr, frontal; P, parietal; T, temporal; I, insula; NA, not available. Participant 11 was removed due to the presence of muscle activity during the slow stretches*.

### Passive Musculoskeletal Stiffness

**Figure 2:**
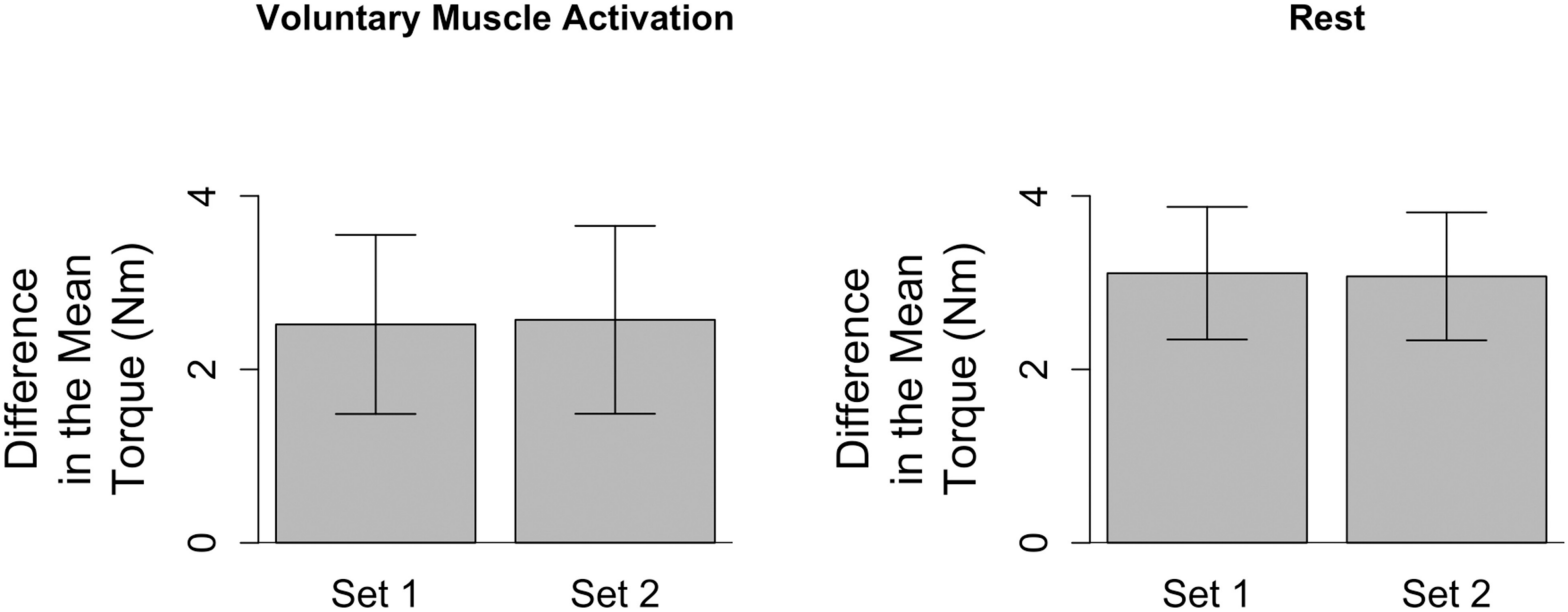
Difference in the mean torque, a proxy for the passive musculoskeletal stiffness, across each session. Mean (bar height) and lower and upper 95th percentile confidence intervals (error bars) are identified for participants' difference in the mean torque during each slow stretching set (Set 1 and Set 2) of every session (voluntary muscle activation and rest).

To address whether the passive musculoskeletal stiffness changed within each testing session, we compared the difference in the mean torque (τ_150°_ − τ_70°_) for the first and second slow stretching set of that testing session (Figure 2). Data for one participant were removed from both testing sessions due to the presence of muscle activity during the slow stretches, as determined from analyses of the sEMG signals. Therefore, the remaining analyses are based on the remaining 16 participants.

During the voluntary muscle activation session, muscle activity was observed for a second participant during the slow stretches, as determined from the sEMG signals; hence, data for this participant were removed from further analyses of this testing session. In turn, future analyses for the voluntary muscle activation session were based on the remaining 15 participants.

Finally, during the rest session, there was a data storage error during the slow stretches of the second set for one participant, and, hence, data for this participant were not included in the analyses relevant to the passive musculoskeletal stiffness. The data analyzed for this participant for the first set of this session, as well as both sets of the voluntary muscle activation session, did not reveal the presence of muscle activity. Hence, while we excluded this participant from the analyses relevant to the passive musculoskeletal stiffness, we did include this participant's data in the remaining analyses for the 120°/s stretches.

Analyses of the passive musculoskeletal stiffness during the slow stretches were based on 15 participants for each session since two participants were excluded from each session, as discussed above. Results shown in Figure 2 indicate that the difference in the mean torque (τ_150°_ − τ_70°_), as defined in section Quantifying Passive Musculoskeletal Stiffness—Slow Stretches, did not significantly change between the first and second set of slow stretches of the voluntary muscle activation [*t*_(14)_ = −0.37; *p* = 0.718] and rest [*t*_(14)_ = 0.06; *p* = 0.951] sessions. Consequently, we conclude that the passive musculoskeletal stiffness remained similar throughout each session, providing support that the outcome measures described in section Quantifying Stretch Reflex Activity—Fast Stretches are reasonable.

### Preparation of Fast Stretch Data for Analyses

For the 120°/s stretching data, separate analyses were run for the different muscle groups (flexors and extensors). That is, when the forearm extended from 70° to 150°, we obtained information about how the stretched flexor muscles responded. Likewise, when the forearm flexed from 150° to 70°, we obtained information about how the stretched extensor muscles responded. Hereafter, results will be presented by the muscle group stretched, i.e., flexors and extensors.

As discussed in section Passive Musculoskeletal Stiffness, data from two participants for the volitional muscle activation session and one participant for the rest session were excluded due to the presence of muscle activity during the slow stretches. Given that the baseline muscle activity existed, we could not clearly determine how the 120°/s stretching impacted these participants' reflex activity. Therefore, results presented for the volitional muscle activation and rest sessions are based on the remaining 15 and 16 participants, respectively.

#### Flexors

We confirmed that muscle flexor activity was present in the first stretch of the first 120°/s stretching set of each testing session for every participant (see section Quantifying Stretch Reflex Activity—Fast Stretches). In this way, we could confirm that attenuation of the stretch reflex activity was occurring. Data for one participant in each testing session were removed due to quiescent flexor muscle activity. Therefore, results for the flexors during the volitional muscle activation and rest sessions are based on 14 and 15 participants, respectively. Results comparing these testing sessions are based on 13 participants for whom data existed in both.

#### Extensors

We confirmed that muscle extensor activity was present in the first stretch of the first 120°/s stretching set of each testing session for every participant (see section Quantifying Stretch Reflex Activity—Fast Stretches). In this way, we could confirm that attenuation of the stretch reflex activity was occurring. Data for nine participants in the volitional muscle activation session and 11 participants in the rest session were removed from the analyses for the extensors due to quiescent extensor muscle activity. Therefore, results for the extensors during the volitional muscle activation session and rest session are based on six and five participants, respectively. Results comparing these sessions are based on four participants for whom data existed in both.

### Impact of Voluntary Muscle Activation and Rest on Reflex Activity Across All Fast Stretches

**Figure 3:**
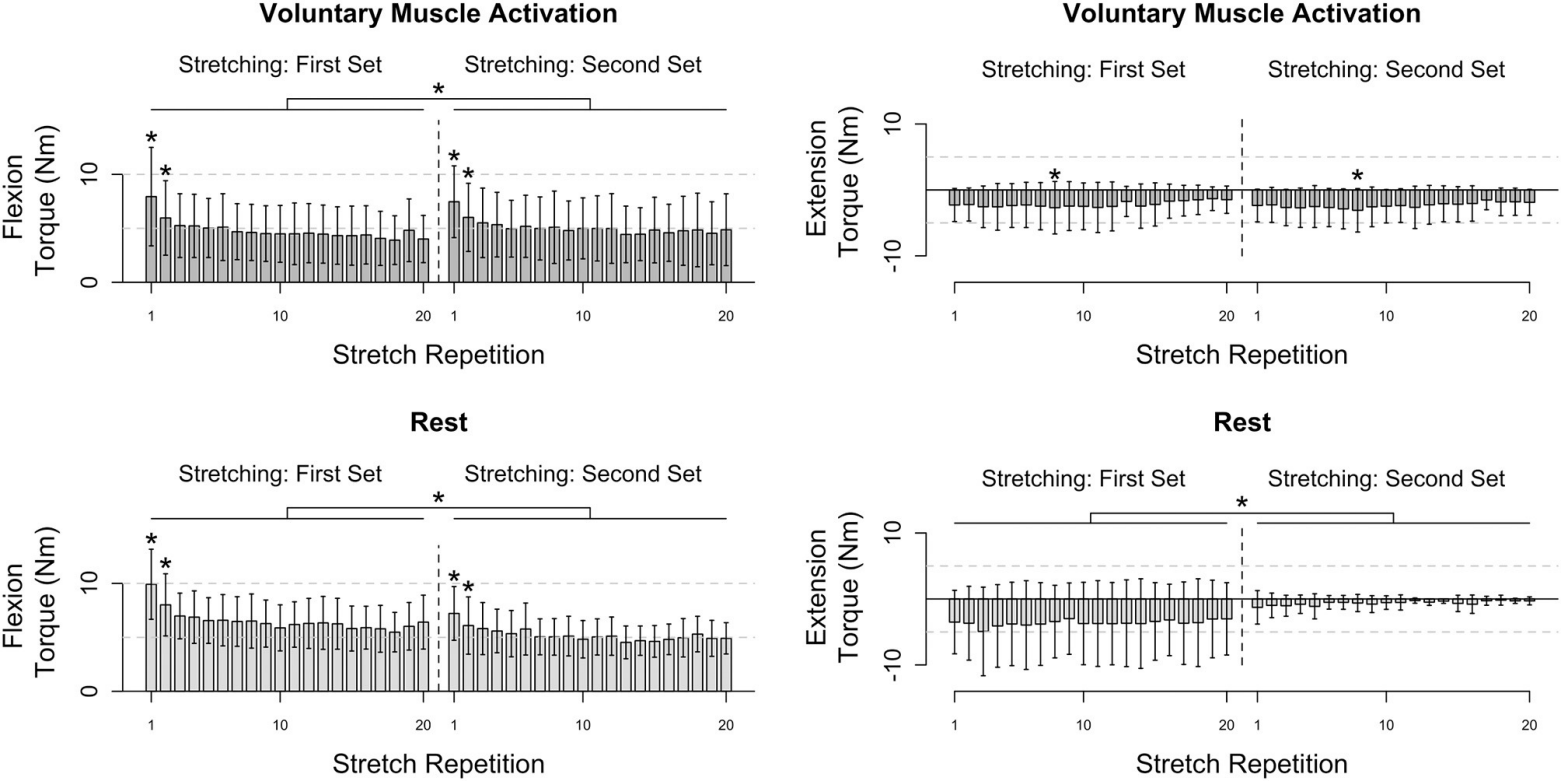
Participants' flexion and extension torque as a function of stretch repetition prior to and following voluntary muscle activation and rest. Mean (bar height) and lower and upper 95th percentile confidence intervals (error bars) are identified. A line with a star above indicates a significant difference between sets. An individual star indicates stretch repetitions that significantly differ from subsequent stretch repetition(s). *Post-hoc* comparisons for each significant stretch repetition are provided in Figure 4.

**Figure 4:**
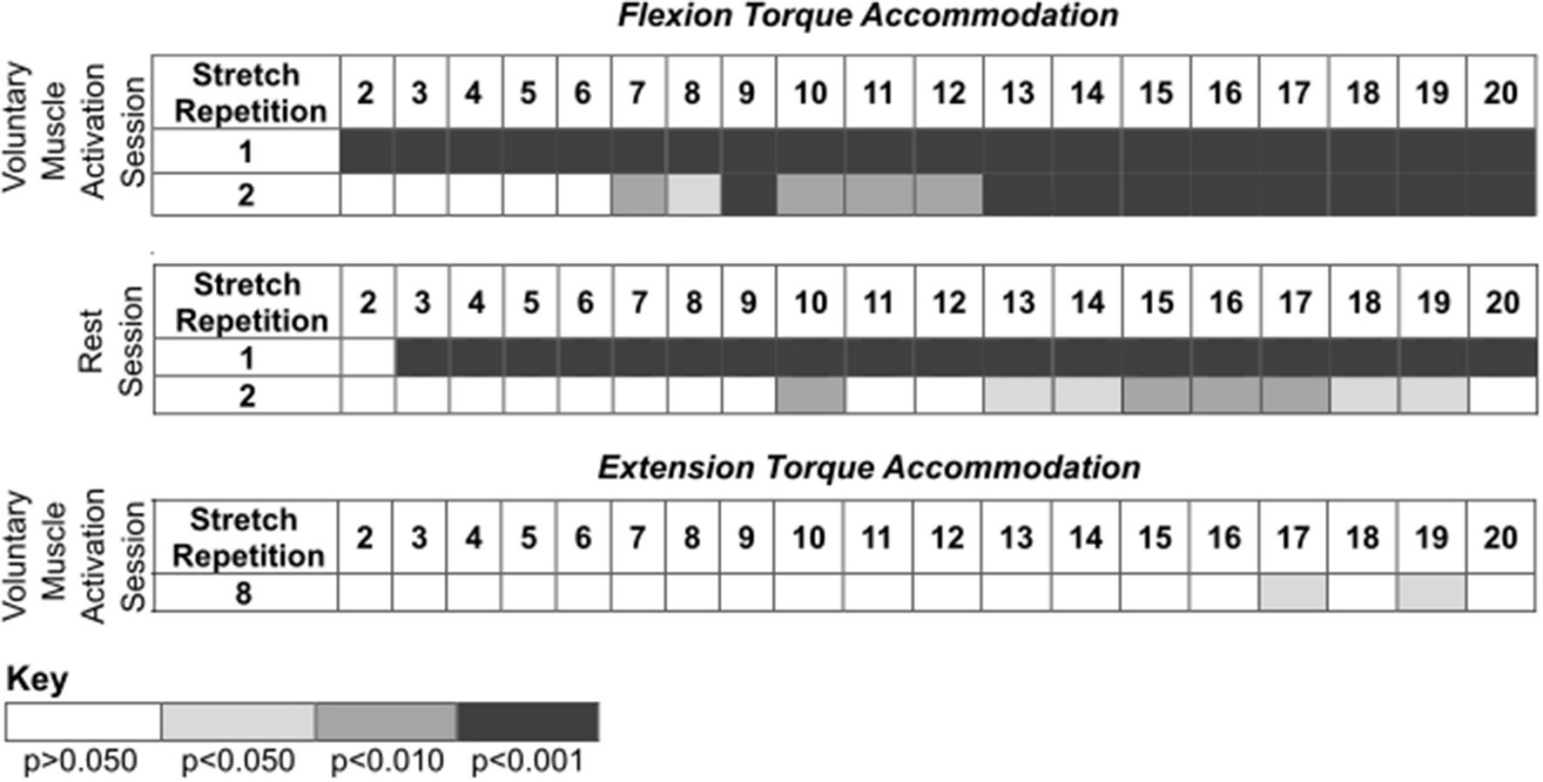
*Post-hoc* comparisons for the results presented in Figure 3 during the voluntary muscle activation and rest sessions. Rows identify the *i*th stretch repetition, and columns identify subsequent stretch repetitions. Rows are not included for stretch repetitions that did not have significance. Significance is represented using shading—white: *p* > 0.050; light gray: *p* < 0.050; darker gray: *p* < 0.010; darkest gray: *p* < 0.001.

Results are summarized in Figures 3, 4.

#### Flexors

During the voluntary muscle activation session, the net torque reduced as the stretch repetition increased [*F*_(19, 526)_ = 14.78; *p* < 0.001], being greater on the first two fast stretches than subsequent stretches (*p* < 0.050). Additionally, the net torque was greater across the second set of 120°/s stretches following voluntary muscle activation than the first set [*F*_(1, 526)_ = 9.64; *p* = 0.002]. Therefore, volitional muscle activation amplified stretch reflex activity across subsequent fast stretches.

During the rest session, the net torque decreased with stretch repetition [*F*_(19, 526)_ = 8.34; *p* < 0.001], being greater on the first two fast stretches than on subsequent stretches (*p* < 0.050). Additionally, the net torque was less across the second set of fast stretches following rest than the first set [*F*_(1, 565)_ = 116.73; *p* < 0.001]. Therefore, the stretch reflex activity was not found to be noticeably affected by the rest.

#### Extensors

During the voluntary muscle activation session, the net torque depended on the stretch repetition [*F*_(19, 214)_ = 2.62; *p* < 0.001], with a significant difference between the eighth and subsequent fast stretches (*p* > 0.050), yet did not depend on the stretching set [*F*_(1, 214)_ = 2.13; *p* = 0.146]. Therefore, the 120°/s stretches did not notably attenuate extensor reflex activity.

During the rest session, the net torque did not significantly depend on the stretch repetition [*F*_(19, 175)_ = 0.11; *p* = 1.000]; yet, the net torque was less across the second fast stretching set following rest than the first [*F*_(1, 175)_ = 56.96; *p* < 0.001]. Therefore, each individual 120°/s stretch did not noticeably attenuate the extensor reflex activity whereas there was a cumulative effect.

### Pairwise Comparisons

**Figure 5:**
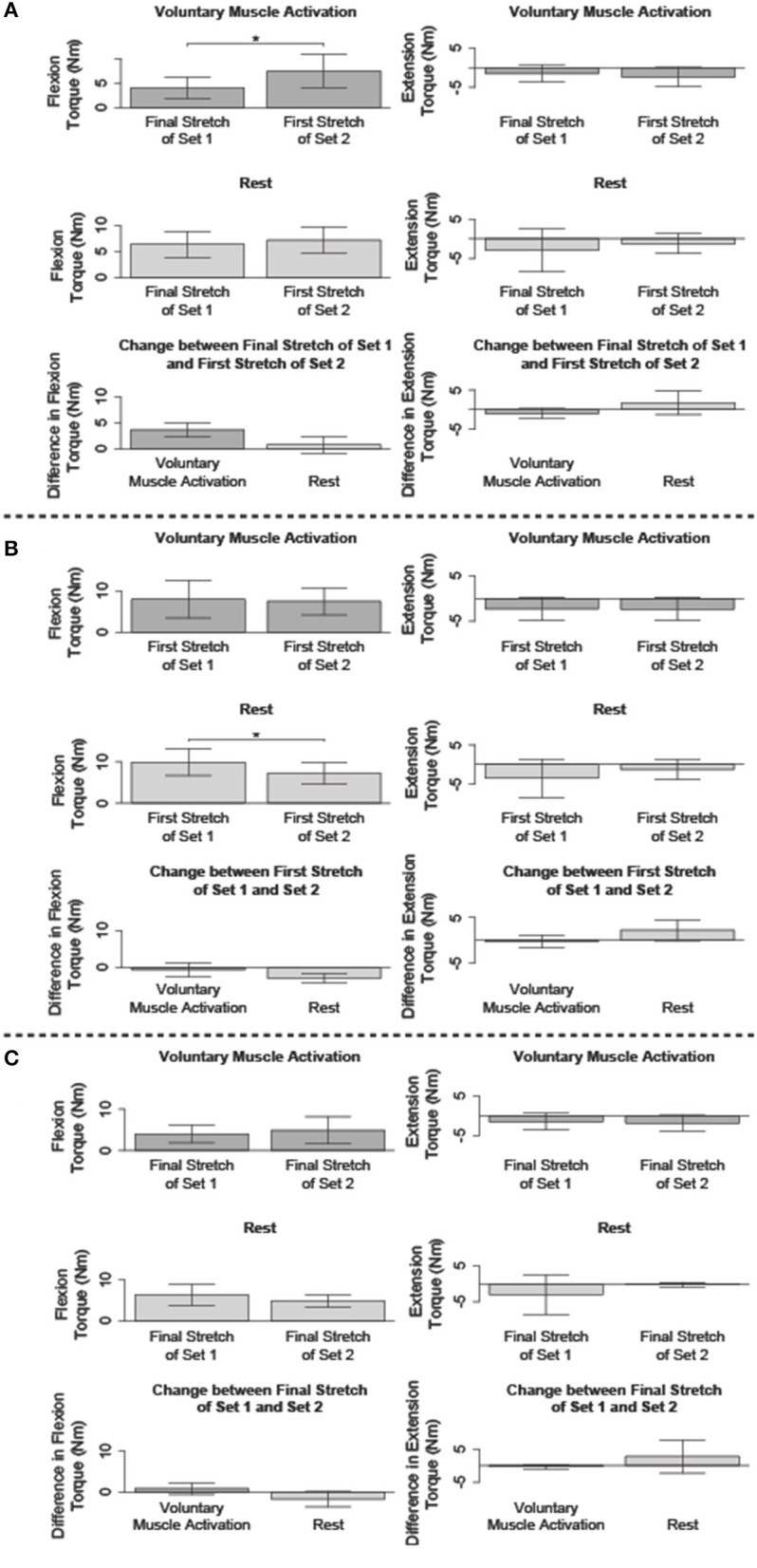
Comparison of the flexion and/or extension torque during specific stretches. Mean (bar height) and lower and upper 95th percentile confidence intervals (error bars) are identified. A line with a star above indicates a significant difference. Comparison between the: **(A)** final fast stretch of set 1 and initial fast stretch of set 2, **(B)** initial fast stretch of set 1 and initial fast stretch of set 2, and **(C)** final fast stretch of set 1 and final fast stretch of set 2.

Figure 5 summarizes the impact of voluntary muscle activation and rest on specific pairs of fast stretches.

#### Immediate Impact of Voluntary Muscle Activation and Rest on Reflex Activity When Compared to Stretch-Attenuated Level

We compared the net torque of the second set's first fast stretch to the first set's final fast stretch to determine the immediate impact of volitional muscle activation and rest on stretch-induced attenuated stretch reflex activity (Figure 5A).

##### Flexors

The net torque increased following voluntary muscle activation [*t*_(13)_ = −5.22; *p* < 0.001], but not rest [*t*_(14)_ = −1.09; *p* = 0.879]. The difference in the net torque between these two fast stretches did not significantly differ between the voluntary muscle activation and rest sessions [*t*_(12)_ = 2.62; *p* = 0.067], albeit there was a trend toward significance. Combined, these results suggest that flexor reflex activity increased immediately with volitional muscle activation, but not rest.

##### Extensors

No significant difference in the net torque was found between these two 120°/s stretches following voluntary muscle activation [*t*_(5)_ = 2.04; *p* = 0.289] and rest [*t*_(4)_ = −1.15; *p* = 0.948]. Therefore, extensor reflex activity was sustained regardless of volitional muscle activation or rest.

#### Immediate Impact of Voluntary Muscle Activation and Rest on Reflex Activity When Compared to Pre-stretching Level

We compared the net torque of the second set's first fast stretch to the first set's first fast stretch to determine the immediate impact of volitional muscle activation and rest on stretch reflex activity when compared to pre-accommodation levels (Figure 5B).

##### Flexors

The net torque from the first 120°/s stretch was greater for the first set than the second set for the rest session [*t*_(14)_ = 4.78; *p* < 0.001], but not the voluntary muscle activation session [*t*_(13)_ = 0.053; *p* = 1.000]. Even so, the difference in the net torque between these two fast stretches did not significantly differ when comparing the voluntary muscle activation and rest sessions [*t*_(12)_ = 2.02; *p* = 0.198]. Combined, these results suggest that volitional muscle activation restored flexor reflex activity to pre-stretch levels whereas rest maintained the stretch-induced flexor reflex accommodation.

##### Extensors

The net torque did not significantly change between these two fast stretches during the voluntary muscle activation [*t*_(5)_ = 0.14; *p* = 1.000] and rest [*t*_(4)_ = −1.91; *p* = 0.388] sessions. Therefore, extensor stretch reflex activity was at a pre-stretch level immediately following volitional muscle activation and rest.

#### Impact of Voluntary Muscle Activation and Rest on Reflex Activity of Final Fast Stretches

We compared the net torque from the final fast stretch of each set to determine whether voluntary muscle activation and rest impacted the level to which our stretching protocol accommodated reflex activity (Figure 5C).

##### Flexors

The net torque did not significantly differ between these two fast stretches during the voluntary muscle activation [*t*_(13)_ = −1.27; *p* = 0.679] and rest [*t*_(14)_ = 1.69; *p* = 0.339] sessions. Therefore, our stretching protocol accommodated flexor stretch reflex activity to similar levels regardless of the testing condition.

##### Extensors

The net torque did not significantly differ between these two fast stretches during the voluntary muscle activation [*t*_(5)_ = 1.99; *p* = 0.954] and rest [*t*_(4)_ = −1.06; *p* = 1.000] sessions. Therefore, our stretching protocol accommodated extensor stretch reflex activity to similar levels regardless of the testing condition.

#### Short- and Long-Latency Reflex Response

Results are summarized in Supplementary Figures 1–3.

##### Flexors

To describe the flexors' SLR and LLR, we analyzed the sEMG data captured from the biceps brachii. Across all fast stretches, the average speed at which the forearm rotated during the time segment corresponding to the SLR and LLR was 94.7°/s and 117.9°/s, respectively. Hence, the spinal contribution to the SLR and LLR could have differed due to the change in the angular speed at which the forearm rotated during each respective time window. Therefore, we cannot draw conclusions regarding contributions arising from the transcortical input since the spinal input was still changing during the LLR time window due to the ramping up of the angular speed.

During the voluntary muscle activation session, the biceps brachii SLR [*F*_(19, 526)_ = 8.28; *p* < 0.001] and LLR [*F*_(19, 526)_ = 8.28; *p* < 0.001] reduced with stretch repetition, with muscle activity being greater on the first fast stretch than subsequent stretches (*p* < 0.050). The stretching set did not significantly affect the SLR [*F*_(1, 526)_ = 0.02; *p* = 0.895] and LLR [*F*_(1, 526)_ = 0.08; *p* = 0.772]. These results indicate that a short-latency response could explain changes in the biceps brachii reflex activity throughout the voluntary muscle activation session; due to the change in the angular speed at which the forearm rotated during the SLR and LLR time windows, the contributions during the long-latency response remain inconclusive.

During the rest session, the biceps brachii SLR [*F*_(19, 565)_ = 1.95; *p* = 0.010] and LLR [*F*_(19, 565)_ = 2.45; *p* < 0.001] reduced with stretch repetition, with muscle activity being greater on the first fast stretch than subsequent stretches (*p* < 0.050). Additionally, the SLR increased from the first to the second fast stretching set [*F*_(1, 565)_ = 4.80; *p* = 0.029], whereas the LLR did not significantly change [*F*_(1, 565)_ = 0.98; *p* = 0.323]. These results, again, indicate that the short-latency response can explain changes in the biceps brachii reflex activity throughout the rest session; due to the change in the angular speed at which the forearm rotated during the SLR and LLR time windows, the contributions during the long-latency response remain inconclusive.

##### Extensors

To describe the extensors' SLR and LLR, we analyzed the sEMG data captured from the lateral head of the triceps brachii. Across all fast stretches, the average speed at which the forearm rotated during the time segment corresponding to the SLR and LLR was 94.5°/s and 118.2°/s, respectively. Hence, the spinal contribution to the SLR and LLR could have differed due to the change in the angular speed at which the forearm rotated during each respective time window. Therefore, we cannot draw conclusions regarding contributions arising from the transcortical input since the spinal input was still changing during the LLR time window due to the ramping up of the angular speed.

During the voluntary muscle activation session, the triceps brachii SLR [*F*_(19, 214)_ = 2.51; *p* < 0.001] and LLR [*F*_(19, 214)_ = 2.45; *p* = 0.001] reduced with stretch repetition, being greater on the eighth fast stretch than the seventeenth fast stretch and nineteenth fast stretch (*p* < 0.050). Additionally, the triceps brachii activity was greater on the second fast stretching set, after volitional muscle activation, than the first set for the SLR [*F*_(1, 214)_ = 72.06; *p* < 0.001] and LLR [*F*_(1, 214)_ = 65.54; *p* < 0.001]. These results suggest that the short-latency response can explain changes in the triceps brachii reflex activity throughout the voluntary muscle activation session; due to the change in the angular speed at which the forearm rotated during the SLR and LLR time windows, the contributions during the long-latency response remain inconclusive.

During the rest session, the triceps brachii activity was less on the second fast stretching set, after rest, than the first fast stretching set for the SLR [*F*_(1, 175)_ = 61.66; *p* < 0.001] and LLR [*F*_(1, 175)_ = 60.94; *p* < 0.001]. The triceps brachii activity was not found to be significantly affected by the stretch repetition for the SLR [*F*_(19, 175)_ = 0.13; *p* = 1.000] and LLR [*F*_(19, 175)_ = 0.13; *p* = 1.000]. These results demonstrate that the short-latency response can explain changes in the triceps brachii reflex activity during the rest session; due to the change in the angular speed at which the forearm rotated during the SLR and LLR time windows, the contributions from the long-latency response remain inconclusive.

## Discussion

We examined whether volitional muscle activation altered stretch reflex activity following consecutive fast stretches in individuals with stroke. To begin, we demonstrated that the fast stretches attenuated stretch reflex activity in the flexor muscles. Following, we showed, for the first time, that subsequent voluntary muscle activation reverses stretch-induced reflex accommodation of the flexors.

The majority of the data for our participants' extensor muscles were excluded due to quiescent extensor muscle activity during the 120°/s stretches; the absence of extensor reflex activity is likely due to the stretching speed of 120°/s not being fast enough (19, 32). Therefore, analyses for the extensors were based on a low number of participants. Prior research corroborates this finding that, in the upper limb, the extensors are not as affected with motor deficits, including spasticity, as the flexors (33–37). Given the limitation of the quiescent muscle activity and, in turn, small sample size for the extensors, we chose to not discuss and draw conclusions based on these data. Therefore, the following discussion only reflects our findings for the elbow flexors.

We also highlight that the mechanism governing stretch-induced reflex accommodation remains unclear; existing literature points to possible neural and mechanical origins (15, 38–40). Hence, we discuss our results in light of these potential reflex-accommodating mechanisms.

### Impact of Voluntary Muscle Activation on Flexor Reflex Activity Following Stretch-Induced Accommodation

Our results indicate that volitional muscle activation restores flexor stretch reflex activity to initial hyperactive levels, despite stretch-induced reflex accommodation. Here, we consider three mechanisms that potentially underly this restoration: (1) motoneuron excitability and monoaminergic drive; (2) spindle afferent activity; and (3) passive musculoskeletal stiffness.

#### Motoneuron Excitability and Monoaminergic Drive

Research shows that descending noradrenergic and serotonergic neural activity, and, subsequently, motoneuron excitability, increases with voluntary movement (18, 41, 42). Noradrenergic neurons increased firing before and during voluntary muscle activation in cats and monkeys, while serotonergic neurons increased firing corresponding to the voluntary motor output in cats (18, 41, 42). Norepinephrine and serotonin are monoamines that heighten motoneuron excitability by increasing resting membrane potential, decreasing firing threshold, and contributing to persistent inward currents (43–45). As hyperactive stretch reflexes arise from increased motoneuron excitability, it follows that increased monoaminergic input with voluntary movement would heighten motoneuron excitability and, thus, stretch reflex activity. Post-stroke, bulbospinal pathways containing these monoaminergic neurons are upregulated, such that this proposed mechanism becomes especially relevant for explaining our results (2, 10–12, 46, 47).

#### Spindle Afferent Activity

Muscle contraction can lead to postcontraction sensory discharge, or a prolonged increased firing rate and dynamic stretch sensitivity in muscle spindles from persistent actin-myosin cross-bonds (48–52). Increased spindle afferent activity and, thus, excitatory input to spinal motoneurons would increase motoneuron excitability and, in turn, stretch reflex activity. Spinal animals exhibit this elevated activity, suggesting supraspinal input is not necessary for increased motoneuron activity post-contraction (53). While spindle discharge rates have been shown to increase with voluntary contraction, research has shown that heightened spindle activity does not contribute to spasticity since individuals with stroke have the same spindle sensitivity as individuals who have not had a stroke (54, 55). Therefore, an increase in spindle afferent activity post voluntary contraction is not a likely contributor to the observed reversal in stretch reflex activity.

#### Passive Musculoskeletal Stiffness

Passive musculotendon stiffness can change with repeated stretching (25–29); however, we did not observe a noticeable change in our proxy outcome measure used to identify the passive musculoskeletal stiffness across the span of each session (see section Passive Musculoskeletal Stiffness). This finding suggests that the underlying mechanism of the reflex accommodation and its reversal is not musculoskeletal in nature.

In summary, our results suggest that volitional muscle activation restores flexor stretch reflex activity to initial hyperactive levels, despite reflex accommodation induced by repeated fast stretches. Changes in motoneuron excitability post voluntary contraction is the most likely contributor to these findings.

### Impact of Rest on Flexor Stretch Reflex Activity Following Stretch-Induced Accommodation

Our results suggest that stretch reflex accommodation is sustained in the absence of volitional muscle activation and that rest does not facilitate noticeable further reflex accommodation with subsequent consecutive 120°/s stretches. This finding corroborates previous research that followed a different stretching protocol and showed that accommodated elbow reflex activity was not restored after 3–5 min of rest (56). The mechanisms proposed above could have been maintained while resting, allowing the decreased stretch reflex activity to be sustained.

### Impact of Voluntary Muscle Activation and Rest on Stretch-Induced Flexor Stretch Reflex Accommodation

The impact of the 120°/s fast consecutive stretches on the flexors was most evident within the first few perturbations. This short-lived efficacy may arise from a consistent elevated tonic monoaminergic supply post-stroke, which limits the level of reduction possible such that reflex activity plateaus (12, 57). As the mechanism underlying stretch-induced accommodation is still uncertain, additional neural mechanisms such as Ia synaptic plasticity and reduced motor neuron excitability following repeated stretch-induced activation could have impacted the level to which the reflex activity accommodated (15, 38–40).

### Short-Latency and Long-Latency Response

While we did not investigate the specific underlying mechanisms of the stretch reflex accomodation, we did analyze the muscle activity over time windows corresponding to the short-latency and long-latency stretch responses associated with spinal and transcortical circuitry, respectively. Our analyses suggest that the short-latency response, associated with the spinal circuitry, contributed to the reflex accommodation and the restoration of heightened stretch reflex activity after volitional muscle activation.

To begin, we observed during the voluntary muscle activation session that the short-latency response decreased with the number of 120°/s stretches, yet was not affected by the stretching set. However, during the rest session the short-latency response decreased with the number of fast stretches, as well as the stretching set. Therefore, it appears that the volitional muscle activity increased the short-latency response such that it was comparable across the entire first set of fast stretches when compared to the entire second set of fast stretches. In contrast, during the rest session the short-latency response was significantly less across the entire second set of 120°/s stretches when compared to the entire first set of fast stretches. Hence, any noticeable increase in the short-latency response due to rest was not observed.

Conclusions about the impact of the voluntary muscle activation and rest on the long-latency response, which is associated with transcortical circuitry, cannot be deduced. This is because the angular speed differed during the time windows corresponding to the short- and long-latency response, potentially contributing to a speed-dependent change in spinal activity that was greater than the change in transcortical activity.

Future work is needed to improve our understanding for the mechanism contributing to the impact of volitional muscle activation on reversing stretch-induced reflex accommodation.

### Limitations

One limitation of this study is that the angular speed of 120°/s did not elicit a stretch reflex in all participants. This speed was selected since it is faster than speeds used in previous research; yet, using even faster speeds may have been more effective for eliciting responses in the flexors as well as extensors of all participants (15). Second, only one stretching protocol was examined in this experiment. Different stretching interventions, including of varying angular range, velocity, repetitions, and frequency, may lead to different results with regards to the rate of reflex accommodation and the impact of volitional muscle activation and rest. Third, only a ballistic movement, selected for its functional relevance, was tested; other voluntary movement types (e.g., slow, isometric) could induce different effects on stretch reflex activity. Fourth, this study examined the immediate impact of voluntary movement on stretch reflex activity without addressing long-term effects. Currently, the bulk of the literature examines the effects of stretching within a single session and neglects the long-term effects. Further research is needed to understand the effect of stretching, and volitional muscle activation after stretching, on spasticity in the long term.

## Concluding Remarks

Our findings indicate that stretch reflex accommodation can be altered by volitional muscle activation. Clinically, our findings suggest that the therapeutic benefit of accommodating stretch reflex activity in individuals with stroke through fast consecutive stretches may be reversed once the individual volitionally activates their paretic arm. This study examined a single, precisely-controlled, robot-mediated stretching protocol, whereas there is a plethora of stretching protocols utilized in the clinical setting that have much greater stretch-to-stretch variation. Further testing is needed to determine if voluntary muscle activation yields similar results with other stretching protocols. Even so, as long as the stretch reflex threshold is reached, the results of the current study are likely to persist. Moreover, additional research is needed to elucidate the mechanism(s) contributing to increased stretch reflex activity post-volitional muscle activation, in addition to determining the exact neural mechanism(s) contributing to the accommodation of the stretch reflex when stretching. For individuals with a unilateral brain injury due to a stroke, who cannot volitionally activate their paretic arm, the stretch-induced reflex accommodation arising from fast consecutive stretching may remain beneficial. To conclude, future work is needed to understand the long-term implications of fast consecutive stretches as an effective treatment for stretch reflex hyperactivity, or spasticity, in individuals with stroke.

## Data Availability Statement

The datasets presented in this study can be found in online repositories. The name of the repository and accession number can be found below: Harvard Dataverse, https://doi.org/10.7910/DVN/ZG9KX8.

## Ethics Statement

The studies involving human participants were reviewed and approved by Northwestern University Institutional Review Board. The participants provided their written informed consent to participate in this study. Written informed consent was obtained from the individual(s) for the publication of any potentially identifiable images or data included in this article.

## Author Contributions

JP, JPAD, JMD, and NG designed the experimental protocol, contributed to the interpretation of the data, and revised the manuscript. NG implemented the experimental protocol on the robotic device. JPAD and NG supervised the research process. JP and JMD conducted the experiments. JMD conducted the clinical assessments. JP and NG analyzed the data and created the original manuscript and figures. All authors contributed to the article and approved the submitted version.

## Funding

Funding for this research was provided by NIH K25HD096116 (PI: NG), R21HD099710 (Co-PI: JPAD; Co-I: NG), and R01HD039343 and R01NS105759 (PI: JPAD).

## Conflict of Interest

The authors declare that the research was conducted in the absence of any commercial or financial relationships that could be construed as a potential conflict of interest.

## Publisher's Note

All claims expressed in this article are solely those of the authors and do not necessarily represent those of their affiliated organizations, or those of the publisher, the editors and the reviewers. Any product that may be evaluated in this article, or claim that may be made by its manufacturer, is not guaranteed or endorsed by the publisher.

## Supplementary Material

Supplementary Figure 1 Participants' short-latency response (SLR) as a function of stretch repetition prior to and following voluntary muscle activation and rest. The responses for the flexors and extensors are identified by the muscle activity of the biceps brachii and triceps brachii, respectively. Mean (bar height) and lower and upper 95th percentile confidence intervals (error bars) are identified. A line with a star above indicates a significant difference between sets. An individual star indicates stretch repetitions that significantly differ from subsequent stretch repetition(s). *Post-hoc* comparisons for each significant stretch repetition are provided in Supplementary Figure 3.

Supplementary Figure 2 Participants' long-latency response (LLR) as a function of stretch repetition prior to and following voluntary muscle activation and rest. The responses for the flexors and extensors are identified by the muscle activity of the biceps brachii and triceps brachii, respectively. Mean (bar height) and lower and upper 95th percentile confidence intervals (error bars) are identified. A line with a star above indicates a significant difference between sets. An individual star indicates stretch repetitions that significantly differ from subsequent stretch repetition(s). *Post-hoc* comparisons for each significant stretch repetition are provided in Supplementary Figure 3.

Supplementary Figure 3 Post-hoc comparisons for the results presented in Supplementary Figures 1, 2 during the voluntary muscle activation and rest sessions. Rows identify the ith stretch repetition, and columns identify subsequent stretch repetitions. Rows are not included for stretch repetitions that didi not have significance. Significance is represented using shading—white: *p* > 0.050; light gray: *p* < 0.050; darker gray: *p* < 0.010; darkest gray: *p* < 0.001.

## References

[1] Lance JW. The control of muscle tone, reflexes, and movement: Robert Wartenbeg Lecture. Neurology. (1980) 30:1303–13. 10.1212/WNL.30.12.1303 7192811

[2] Li S. Spasticity, motor recovery, and neural plasticity after stroke. Front Neurol. (2017) 8:120. 10.3389/fneur.2017.00120 28421032 PMC5377239

[3] McPherson JG Stienen AHA Schmit BD Dewald JPA. Biomechanical parameters of the elbow stretch reflex in chronic hemiparetic stroke. Exp Brain Res. (2019) 237:121–35. 10.1007/s00221-018-5389-x 30353212 PMC6402810

[4] Sommerfeld DK Eek EU-B Svensson A-K Holmqvist LW von Arbin MH. Spasticity after stroke: its occurrence and association with motor impairments and activity limitations. Stroke. (2004) 35:134–9. 10.1161/01.STR.0000105386.05173.5E 14684785

[5] Urban PP Wolf T Uebele M Marx JJ Vogt T Stoeter P. Occurence and clinical predictors of spasticity after ischemic stroke. Stroke. (2010) 41:2016–20. 10.1161/STROKEAHA.110.581991 20705930

[6] Wissel J Schelosky LD Scott J Christe W Faiss JH Mueller J. Early development of spasticity following stroke: a prospective, observational trial. J Neurol. (2010) 257:1067–72. 10.1007/s00415-010-5463-1 20140444 PMC2892615

[7] Zorowitz RD Gillard PJ Brainin M. Poststroke spasticity: sequelae and burden on stroke survivors and caregivers. Neurology. (2013) 80:S45–52. 10.1212/WNL.0b013e3182764c86 23319485

[8] Brown P. Pathophysiology of spasticity. J Neurol Neurosurg Psychiatry. (1994) 57:773–7. 10.1136/jnnp.57.7.773 8021658 PMC1073012

[9] Li S Chang S-H Francisco GE Verduzco-Gutierrez M. Acoustic startle reflex in patients with chronic stroke at different stages of motor recovery: a pilot study. Top Stroke Rehabil. (2014) 21:358–70. 10.1310/tsr2104-358 25150668

[10] Li S Chen Y-T Francisco GE Zhou P Rymer WZ. A unifying pathophysiological account for post-stroke spasticity and disordered motor control. Front Neurol. (2019) 10:468. 10.3389/fneur.2019.00468 31133971 PMC6524557

[11] McPherson JG Ellis MD Heckman CJ Dewald JPA. Evidence for increased activation of persistent inward currents in individuals with chronic hemiparetic stroke. J Neurophysiol. (2008) 100:3236–43. 10.1152/jn.90563.2008 18829849 PMC2604864

[12] McPherson JG McPherson LM Thompson CK Ellis MD Heckman CJ Dewald JPA. Altered neuromodulatory drive may contribute to exaggerated tonic vibration reflexes in chronic hemiparetic stroke. Front Hum Neurosci. (2018) 12:131. 10.3389/fnhum.2018.00131 29686611 PMC5900019

[13] Mottram CJ Wallace CL Chikando CN Rymer WZ. Origins of spontaneous firing of motor units in the spastic-paretic biceps brachii muscle of stroke survivors. J Neurophysiol. (2010) 104:3168–79. 10.1152/jn.00463.2010 20861443 PMC3007638

[14] Naro A Leo A Russo M Casella C Buda A Crespantini A. Breakthroughs in the spasticity management: are non-pharmacological treatments the future? J Clin Neurosci. (2017) 39:16–27. 10.1016/j.jocn.2017.02.044 28262404

[15] Schmit BD Dewald JPA Rymer WZ. Stretch reflex adaptation in elbow flexors during repeated passive movements in unilateral brain-injured patients. Arch Phys Med Rehabil. (2000) 81:10. 10.1016/S0003-9993(00)90070-4 10724069

[16] Triandafilou KM Ochoa J Kang X Fischer HC Stoykov ME Kamper DG. Transient impact of prolonged versus repetitive stretch on hand motor control in chronic stroke. Top Stroke Rehabil. (2011) 18:316–24. 10.1310/tsr1804-316 21914596

[17] Vecchio M Gracies J-M Panza F Fortunato F Vitaliti G Malaguarnera G. Change in coefficient of fatigability following rapid, repetitive movement training in post-stroke spastic paresis: a prospective open-label observational study. J Stroke Cerebrovasc Dis. (2017) 26:2536–40. 10.1016/j.jstrokecerebrovasdis.2017.05.046 28666805

[18] Pavlenko VB Kulichenko AM. Self-initiated motor behavioral act-related neuronal activity in the cat locus coeruleus. Neurophysiology. (2003) 35:29–37. 10.1023/A:1023994205918

[19] McPherson JG Stienen AH Drogos JM Dewald JP. Modification of spastic stretch reflexes at the elbow by flexion synergy expression in individuals with chronic hemiparetic stroke. Arch Phys Med Rehabil. (2018) 99:491–500. 10.1016/j.apmr.2017.06.019 28751255 PMC6779818

[20] Heckman CJ Mottram C Quinlan K Theiss R Schuster J. Motoneuron excitability: the importance of neuromodulatory inputs. Clin Neurophysiol. (2009) 120:2040–54. 10.1016/j.clinph.2009.08.009 19783207 PMC7312725

[21] Lincoln N Jackson J Adams S. Reliability and revision of the Nottingham Sensory Assessment for stroke patients. Physiotherapy. (1998) 84:358–65. 10.1016/S0031-9406(05)61454-X

[22] Bovend'Eerdt TJ Newman M Barker K Dawes H Minelli C Wade DT. The effects of stretching in spasticity: a systematic review. Arch Phys Med Rehabil. (2008) 89:1395–406. 10.1016/j.apmr.2008.02.015 18534551

[23] Condliffe EG Clark DJ Patten C. Reliability of elbow stretch reflex assessment in chronic post-stroke hemiparesis. Clin Neurophysiol. (2005) 116:1870–8. 10.1016/j.clinph.2005.02.030 15979400

[24] Levin MF Feldman AG. The role of stretch reflex threshold regulation in normal and impaired motor control. Brain Res. (1994) 657:23–30. 10.1016/0006-8993(94)90949-0 7820623

[25] Herda TJ Ryan ED Costa PB Walter AA Hoge KM Uribe BP. Acute effects of passive stretching and vibration on the electromechanical delay and musculotendinous stiffness of the plantar flexors. Electromyogr Clin Neurophysiol. (2010) 50:277–88. 21061774

[26] Kubo K Kanehisa H Kawakami Y Fukunaga T. Influence of static stretching on viscoelastic properties of human tendon structures *in vivo*. J Appl Physiol. (2001) 90:520–7. 10.1152/jappl.2001.90.2.520 11160050

[27] Kubo K Kanehisa H Fukunaga T. Effects of transient muscle contractions and stretching on the tendon structures *in vivo*. Acta Physiol Scand. (2002) 175:157–64. 10.1046/j.1365-201X.2002.00976.x 12028136

[28] Ryan ED Beck TW Herda TJ Hull HR Hartman MJ Costa PB. The time course of musculotendinous stiffness responses following different durations of passive stretching. J Orthop Sports Phys Ther. (2008) 38:632–9. 10.2519/jospt.2008.2843 18827325

[29] Taylor DC Brooks DE Ryan JB. Viscoelastic characteristics of muscle: passive stretching versus muscular contractions. Med Sci Sports Exerc. (1997) 29:1619–24. 10.1097/00005768-199712000-00011 9432095

[30] Dewald JPA Given JD Rymer WZ. Long-lasting reductions of spasticity induced by skin electrical stimulation. IEEE Trans Rehabil Eng. (1996) 4:231–42. 10.1109/86.547923 8973949

[31] Kamper DG Harvey RL Suresh S Rymer WZ. Relative contributions of neural mechanisms versus muscle mechanics in promoting finger extension deficits following stroke. Muscle Nerve. (2003) 28:309–18. 10.1002/mus.10443 12929190

[32] Powers RK Campbell DL Rymer WZ. Stretch reflex dynamics in spastic elbow flexor muscles. Ann Neurol. (1989) 25:32–42. 10.1002/ana.410250106 2913927

[33] Davidson AG Schieber MH Buford JA. Bilateral spike-triggered average effects in arm and shoulder muscles from the monkey pontomedullary reticular formation. J Neurosci. (2007) 27:8053–8. 10.1523/JNEUROSCI.0040-07.2007 17652596 PMC6672715

[34] Davidson AG Buford JA. Bilateral actions of the reticulospinal tract on arm and shoulder muscles in the monkey: stimulus triggered averaging. Exp Brain Res. (2006) 173:25–39. 10.1007/s00221-006-0374-1 16506008

[35] Kably B Drew T. Corticoreticular pathways in the cat. I Projection patterns and collaterization. J Neurophysiol. (1998) 80:389–405. 10.1152/jn.1998.80.1.389 9658059

[36] Miller LC Dewald JPA. Involuntary paretic wrist/finger flexion forces and EMG increase with shoulder abduction load in individuals with chronic stroke. Clin Neurophysiol. (2012) 123:1216–25. 10.1016/j.clinph.2012.01.009 22364723 PMC3729226

[37] Zaaimi B Edgley SA Soteropoulos DS Baker SN. Changes in descending motor pathway connectivity after corticospinal tract lesion in macaque monkey. Brain. (2012) 135:2277–89. 10.1093/brain/aws115 22581799 PMC3381720

[38] Guissard N Duchateau J. Neural aspects of muscle stretching. Exerc Sport Sci Rev. (2006) 34:154–8. 10.1249/01.jes.0000240023.30373.eb 17031252

[39] Smania N Picelli A Daniele M Geroin C Ianes P Waldner A. Rehabilitation procedures in the management of spasticity. Eur J Phys Rehabil Med. (2010) 46:423–38. 20927008

[40] Tsai K-H Yeh C-Y Chang H-Y Chen J-J. Effects of a single session of prolonged muscle stretch on spastic muscle of stroke patients. Proc Natl Sci Council. (2001) 25:76–81. 11370763

[41] Jacobs BL Martin-Cora FJ Fornal CA. Activity of medullary serotonergic neurons in freely moving animals. Brain Res Rev. (2002) 40:45–52. 10.1016/S0165-0173(02)00187-X 12589905

[42] Wei K Glaser JI Deng L Thompson CK Stevenson IH Wang Q. Serotonin affects movement gain control in the spinal cord. J Neurosci. (2014) 34:12690–700. 10.1523/JNEUROSCI.1855-14.2014 25232107 PMC4166156

[43] Fedirchuk B Dai Y. Monoamines increase the excitability of spinal neurones in the neonatal rat by hyperpolarizing the threshold for action potential production. J Physiol. (2004) 557:355–61. 10.1113/jphysiol.2004.064022 15090607 PMC1665108

[44] Heckman CJ Lee RH Brownstone RM. Hyperexcitable dendrites in motoneurons and their neuromodulatory control during motor behavior. Trends Neurosci. (2003) 26:688–95. 10.1016/j.tins.2003.10.002 14624854

[45] Heckman CJ Johnson M Mottram C Schuster J. Persistent inward currents in spinal motoneurons and their influence on human motoneuron firing patterns. Neuroscientist. (2008) 14:264–75. 10.1177/1073858408314986 18381974 PMC3326417

[46] Fisher KM Zaaimi B Baker SN. Reticular formation responses to magnetic brain stimulation of primary motor cortex. J Physiol. (2012) 590:4045–60. 10.1113/jphysiol.2011.226209 22674723 PMC3464356

[47] McPherson JG Chen A Ellis MD Yao J Heckman CJ Dewald JPA. Progressive recruitment of contralesional cortico-reticulospinal pathways drives motor impairment post stroke. J Physiol. (2018) 596:1211–25. 10.1113/JP274968 29457651 PMC5878212

[48] Brown MC Goodwin GM Matthews PB. The persistence of stable bonds between actin and myosin filaments of intrafusal muscle fibers following their activation. J Physiol. (1970) 210:9. 5500826

[49] Eldred E Hutton RS Smith JL. Nature of the persisting changes in afferent discharge from muscle following its contraction. In: Homma S, editor, Progress in Brain Research. Vol. 44, Progress in Brain Research (1976) p. 157–70. 10.1016/S0079-6123(08)60731-1 137421

[50] Enoka RM Hutton RS Eldred E. Changes in excitability of tendon tap and Hoffmann reflexes following voluntary contractions. Electroencephalogr Clin Neurophysiol. (1980) 48:664–72. 10.1016/0013-4694(80)90423-X 6155255

[51] Gregory JE Mark RF Morgan DL Patak A Polus B Proske U. Effects of muscle history on the stretch reflex in cat and man. J Physiol. (1990) 424:93–107. 10.1113/jphysiol.1990.sp018057 2391663 PMC1189803

[52] Hutton RS Smith JL Eldred E. Postcontraction sensory discharge from muscle and its source. J Neurophysiol. (1973) 36:1090–103. 10.1152/jn.1973.36.6.1090 4271542

[53] Hutton RS Suzuki S. Postcontraction discharge of motor neurons in spinal animals. Exp Neurol. (1979) 64:567–78. 10.1016/0014-4886(79)90232-2 467550

[54] Sheean G. The pathophysiology of spasticity. Eur J Neurol. (2002) 9:3–9. 10.1046/j.1468-1331.2002.0090s1003.x 11918643

[55] Wilson LR Gandevia SC Inglis JT Gracies J-M Burke D. Muscle spindle activity in the affected upper limb after a unilateral stroke. Brain. (1999) 122:2079–88. 10.1093/brain/122.11.2079 10545393

[56] Turpin NA Levin MF Feldman AG. Implicit learning and generalization of stretch response modulation in humans. J Neurophysiol. (2016) 115:3186–94. 10.1152/jn.01143.2015 27052586 PMC4946602

[57] Lee RH Heckman CJ. Enhancement of bistability in spinal motoneurons *in vivo* by the noradrenergic α1 agonist methoxamine. J Neurophysiol. (1999) 81:2164–74. 10.1152/jn.1999.81.5.2164 10322057

