# Supplementary figures and images for: Impact of Voluntary Muscle Activation on Stretch Reflex Excitability in Individuals With Hemiparetic Stroke
Source: Front Neurol. 2022 Mar 8;13:764650. doi: 10.3389/fneur.2022.764650 (PMC8964046; doi:10.3389/fneur.2022.764650)

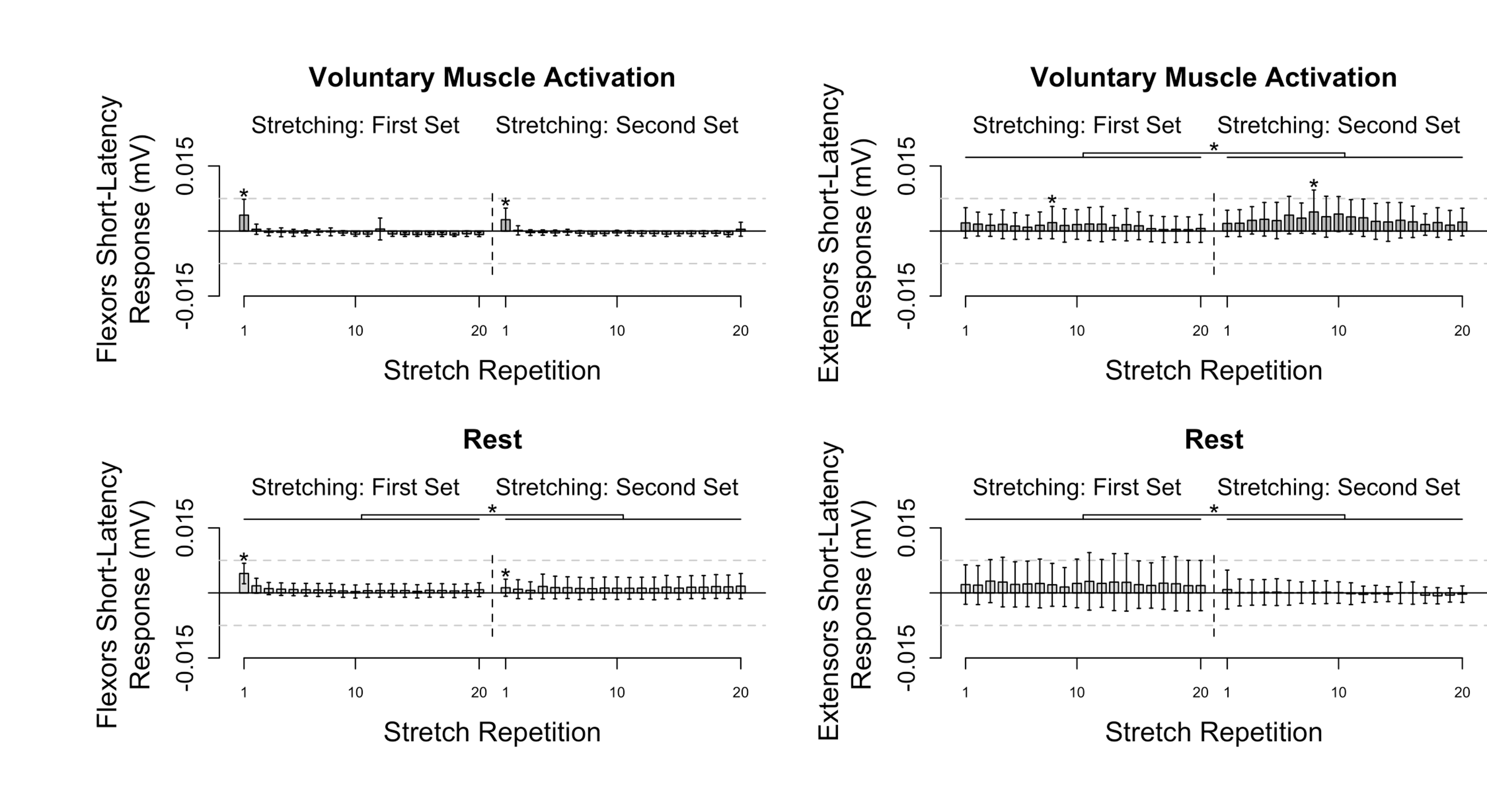

Supplement: Supplementary Figure 1 — Participants' short-latency response (SLR) as a function of stretch repetition prior to and following voluntary muscle activation and rest. The responses for the flexors and extensors are identified by the muscle activity of the biceps brachii and triceps brachii, respectively. Mean (bar height) and lower and upper 95th percentile confidence intervals (error bars) are identified. A line with a star above indicates a significant difference between sets. An individual star indicates stretch repetitions that significantly differ from subsequent stretch repetition(s). Post-hoc comparisons for each significant stretch repetition are provided in Supplementary Figure 3. [file Image_1.TIFF]

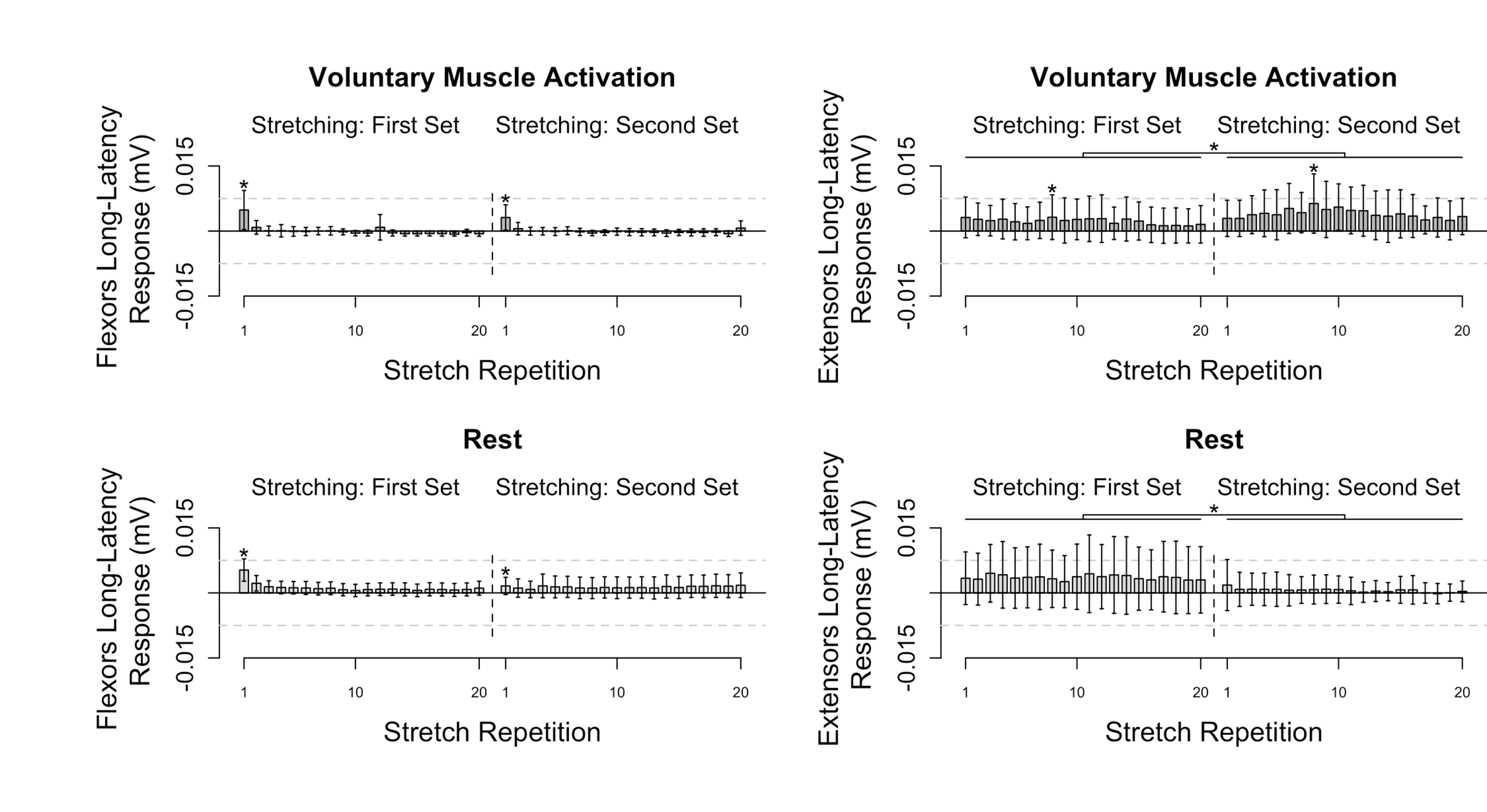

Supplement: Supplementary Figure 2 — Participants' long-latency response (LLR) as a function of stretch repetition prior to and following voluntary muscle activation and rest. The responses for the flexors and extensors are identified by the muscle activity of the biceps brachii and triceps brachii, respectively. Mean (bar height) and lower and upper 95th percentile confidence intervals (error bars) are identified. A line with a star above indicates a significant difference between sets. An individual star indicates stretch repetitions that significantly differ from subsequent stretch repetition(s). Post-hoc comparisons for each significant stretch repetition are provided in Supplementary Figure 3. [file Image_2.TIFF]

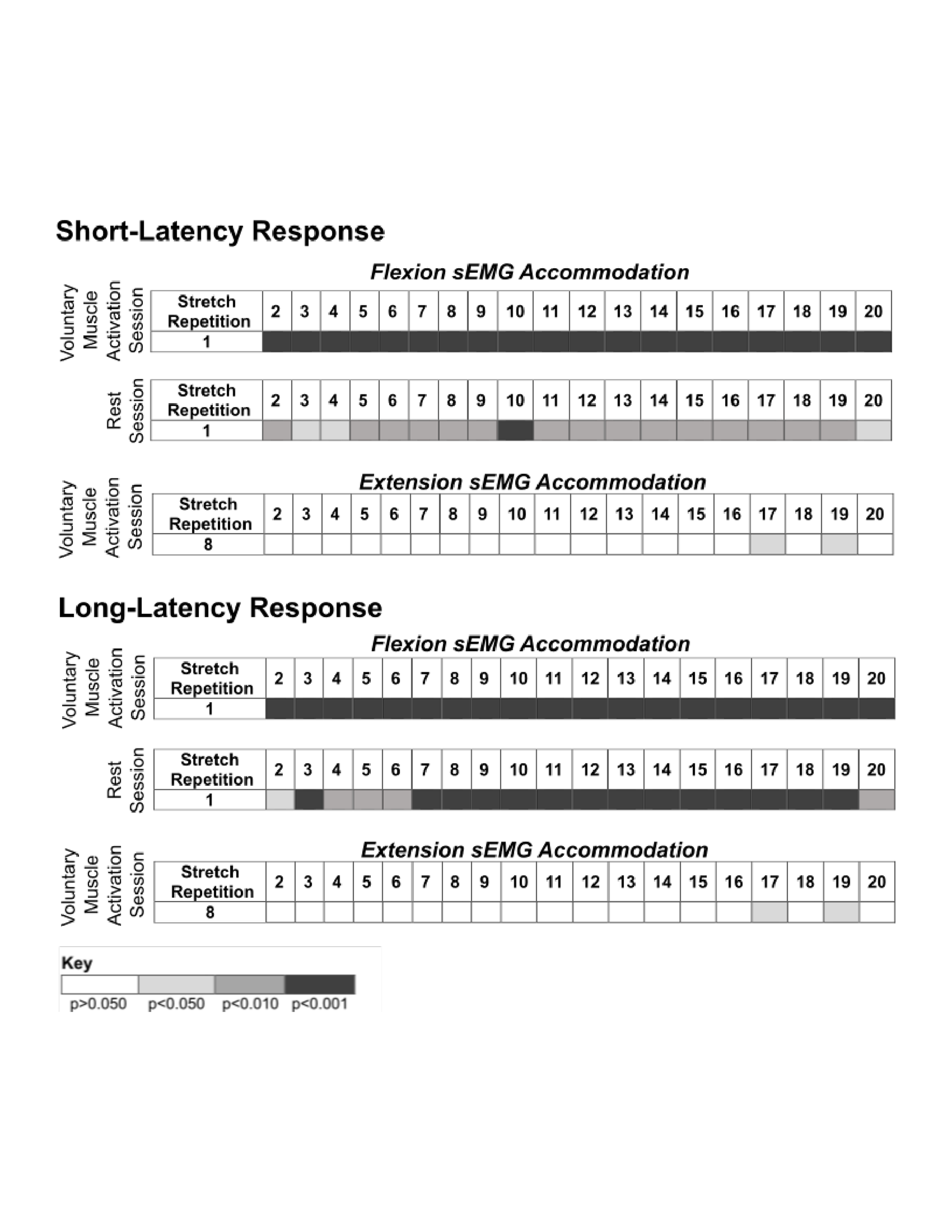

Supplement: Supplementary Figure 3 — Post-hoc comparisons for the results presented in Supplementary Figures 1, 2 during the voluntary muscle activation and rest sessions. Rows identify the ith stretch repetition, and columns identify subsequent stretch repetitions. Rows are not included for stretch repetitions that didi not have significance. Significance is represented using shading—white: p > 0.050; light gray: p < 0.050; darker gray: p < 0.010; darkest gray: p < 0.001. [file Image_3.TIFF]
